# Structure and Properties Study of PA6 Nanocomposites Flame Retarded by Aluminium Salt of Diisobutylphosphinic Acid and Different Organic Montmorillonites

**DOI:** 10.3390/polym10030312

**Published:** 2018-03-13

**Authors:** Wen-Tao He, Sheng-Tao Liao, Yu-Shu Xiang, Li-Juan Long, Shu-Hao Qin, Jie Yu

**Affiliations:** 1 National Engineering Research Center for Compounding and Modification of Polymeric Materials, Guiyang 550014, China; wentao.he@usq.edu.au (W.-T.H.); xiang_ys@126.com (Y.-S.X); longlijuanhappy@126.com (L.-J.L.); 2 Center for Future Materials, University of Southern Queensland, Toowoomba 4350, Australia; 3 College of Material and Metallurgy, Guizhou University, Guiyang 550025, China; liaosthl@163.com

**Keywords:** polyamide 6, organic montmorillonite, nanocomposite, flammability, rheology

## Abstract

Two different types of organic montmorillonite, namely quaternary ammonium salt intercalated MMT (CMMT) and quaternary phosphonium salt intercalated MMT (PMMT) were used as fillers in the flame-retardant polyamide (PA6) based on aluminium salts of diisobutylphosphinic acid (ABPA). The influence of different types of organic montmorillonite (OMMT) on the structure and properties of flame-retardant PA6 nanocomposites were systematically investigated. The X-ray diffraction and transmission electron microscopy results suggested that the introduction of OMMT improved the dispersion of the flame retardant particles independently of the type of OMMT. The derivative thermogravimetry (DTG) curve transformed to one peak from two peaks (representing the degradation of ABPA and PA6, respectively) after incorporation of the OMMT, which further confirmed better ABPA dispersion. Viscoelastic measurements demonstrated that a mechanically stable network structure was formed with the introduction of OMMT or ABPA and OMMT, while PA6/ABPA/PMMT presented the highest storage modulus and viscosity, suggesting a more efficient network structure. From UL-94 and limited oxygen index (LOI) tests, PA6/ABPA/PMMT presented the best flame performance, with a UL-94 of V-0 and a LOI of 33%. In addition, the PA6/ABPA/PMMT presented the lowest peak heat release rate (pHRR) among the investigated samples. Combined with the char layer analysis, it can be deduced that the introduction of PMMT improved the dispersion of ABPA, and promoted the formation of more efficient network structure, before promoting more compact char structures, which finally resulted in improved flame retardancy.

## 1. Introduction

Apart from dramatic improvements in mechanical, thermal, and barrier properties, polymer/clay nanocomposites have been rendered even more attractive by recent demonstrations of their flame-retardant properties, which is namely diminution of the heat release rate. This effect is mainly attributed to the formation of a char barrier on the surface of the polymer, which insulates the underlying materials and slows the mass loss rate of decomposition products during pyrolysis and combustion [1,2]. However, clays have failed to act as stand-alone flame retardants due to only having minor effects in reducing the total heat release, since the nanocomposites do not self-extinguish even after a protective char is formed until most of the fuel has burnt out. Combination of the clay nanocomposites with other flame retardant additives is generally necessary to pass regulatory flammability tests, and can give a final product which has a better balance of mechanical properties and flame retardant properties [3,4,5,6,7,8,9,10]. The synergism between the flame retardant additives and organoclay that improves the flame retardancy has been well documented [11,12]. The improved flame retardancy is generally ascribed to the combined effect of the gas phase from the flame retardant and reinforced condensed phase action by the organoclay. However, few studies have focused on the effect of the microstructure of the flame-retardant nanocomposites [12], such as dispersion and location of organoclay and flame-retardant additives, on the structure of char layer and subsequently the final flame retardancy.

Regarding polymer nanocomposites, previous studies have shown that there is strong relationship between the viscoelastic parameters (storage modulus and the melt viscosity), which can characterize the microstructure of nanocomposites together with X-ray diffraction (XRD) and transmission electron microscopy (TEM), and the nanocomposite flammability [13,14]. The formation of a network structure within a polymer matrix, reflected from viscoelastic curves, generally contributes to significant flammability reduction [15,16]. Kashiwagi et al. investigated the effect of viscoelastic properties of clay and carbon nanocomposites on the flammability. Continuous protective char layers are observed for carbon nanotube nanocomposites, but the clay-particle layers for clay nanocomposites tend to develop large lateral surface cracks. It is proposed that if more stable networks without forming any cracks could be formed during burning, the flammability properties of the clay-based polymer nanocomposites could be further improved [17]. According to Kashiwagi, et al., one possible approach might be reinforcing the network with char using char forming resins. Fang et al. also correlated the clay network structure and flammability properties of ABS/clay and ABS-*g*-MAH/clay nanocomposites. It was found that a clay network structure was formed in ABS-*g*-MAH/clay nanocomposites. The clay network improved the melt viscosity and limited the mobility of the polymer chains during combustion, finally leading to significantly improved flame retardancy for the nanocomposites [18]. To sum up, the flame retardancy of nanocomposites is strongly related to the microstructure and studies focusing on the effect of microstructure on the flame retardancy can provide an important guidance for developing novel flame retardant nanocomposites.

In our previous work, OMMT was introduced in conjunction with the aluminium salt of diisobutylphosphinic acid (ABPA) for preparation of flame-retardant PA6 nanocomposites [19]. According to Yao et al., ABPA was more efficient than aluminium salt of diethylphosphinic acid (AlPi, a commercial flame retardant) for the flame retardancy of nylon at the same loading amount due to its high gas phase activity [20]. Our preliminary experiments also confirmed that ABPA showed superior flame retardancy in PA6 than AlPi (see Supplementary Materials). Further investigations are in progress in our laboratory to explain this. It was expected that the introduction of OMMT would increase the melt viscosity and promote the formation of compact and coherent char build-up on the surface of PA6 flame retarded by ABPA during burning, finally resulted in enhanced flame-retardant activity in the condensed phase. It has been shown that nanocomposites based on nanoclay and flame-retardant particles formed a continuous network structure in the initial samples, before formation a protective solid layer on the burning surface made of clay and carbonaceous material. Compared to the PA6/clay nanocomposites, a more compact protective layer was formed in the case of flame-retardant PA6/clay nanocomposites.

In this study, we examined whether such a relationship can be generalized to flame-retardant polymer nanocomposites based on different types of organic montmorillonite and explored how the different types of OMMT influence the microstructure of the flame retardant nanocomposites and the flame retardancy.

## 2. Experimental Section 

### 2.1. Materials

PA6 (1013B), was manufactured by UBE Co. Ltd. (Tokyo, Japan). AlPi (Exolit OP1230) was purchased from Clariant Co. (Frankfurt, Germany). The organoclay, expressed as CMMT (I.30P), which was modified with octadecylamine, was purchased from Nanocor, Inc. (Arlington Heights, IN, USA. Another organoclay, expressed as PMMT, was prepared by our laboratory [21]. The preparation process is briefly introduced as follows: the dried sodium (Na^+^) MMT (PGN, purchased from Nanocor, Inc.) with a quantitative surfactant (tetradecyltrihexylphosphonium chloride, 0.8 CEC of MMT) were poured into a 1 L high pressure reactor. After the mixture was stirred in scCO_2_ for 2 h at 40 °C and 20 MPa, the system was rapidly depressurized to ambient pressure. Following this, the product was further washed with deionized water several times until no chlorine ions were detected using a 0.1 N AgNO_3_ solution. The organophosphorus flame retardant ABPA was obtained from our laboratory as our previous report [19].

### 2.2. Sample Preparation

**Scheme 1:**
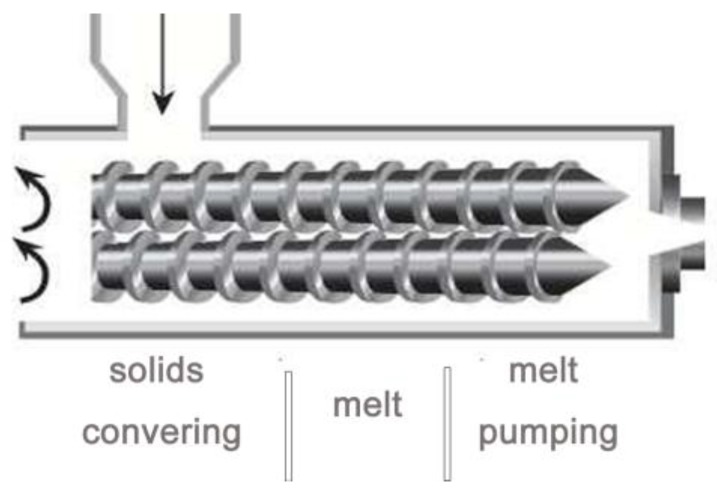
The model of the twin screw extruder.

The PA6, ABPA and organoclays were dried in a vacuum oven at 80 °C for 24 h before mixing. After this, these samples were mixed according to the compositions of composites listed in Table 1 and were prepared by melt compounding in an intermeshing co-rotating twin screw extruder (CTE 20, Coperion Keya Machinery Manufacturing Co., Ltd., Nanjing, China) at the screw speed of 300 r/min, feeding speed of 16 r/min, and processing temperature about 230 °C. The model of the extrude is given is Scheme 1. After that, the extrudates were cut into pellets and were dried at 80 °C for 4 h to remove any water. The dried pellets were further injection-molded using an injection molding machine (CJ80MZ2NCII, Zhende Plastic Machinery Factory, Foshan, China) at the mold temperature of 50–80 °C and melt temperature of 217–237 °C in order to obtain the standard testing bars.

### 2.3. Characterization

XRD was carried out to assess the clay intergallery spacing. Clay powder or composite samples were analyzed by utilizing a D/max 2200 X-ray generator (Rigaku, Tokyo, Japan) with Cu Kα radiation (λ = 0.154 nm, operated at an accelerating voltage of 40 kV and a current of 30 mA). All tests were performed in the reflection mode at ambient temperature with 2°/min varying between from 2° to 10°. TEM was used to represent the dispersion of clay or retardant particles in PA6 composites under the same magnification. The samples for TEM observation were cut into ultrathin sections with thicknesses of 60–80 nm using a microtome, and then they were collected on a 200 mesh carbon film coated copper grid. The TEM images were obtained by a JEM-200CX TEM (JEOL Ltd., Tokyo, Japan) operated at 200 kV.

The rheological properties of samples were measured by means of Rheometric Analyzer (HAAKE MARSII, Thermo Fisher Scientific Inc., Newington, Germany) using parallel plates with diameters of 35 mm. Dynamic frequency sweep tests at 230 °C using 1% strain were investigated in an angular frequency range from 0.01 rad/s to 100 rad/s. The dynamic temperature sweep tests were conducted in the temperature range of 200–300 °C with a heating rate of 5 °C/min at 0.1 rad/s.

Contact angle measurements were performed at ambient temperature using sessile drop method on an DSA 25 contact angle meter (Kruss AG, Hamburg, Germany). The flat disks were compacted from organoclay powders by the tablet press, while the virgin PA6 bas with smooth surfaces were used. The surface behavior of the samples was measured by measurements of contact angles against distilled water and diiodomethane with five replicates respectively. Then, the surface energy, its dispersion and polar forces of samples were calculated according to Young’s Equation (1), in addition, combined with Equation (2) and Wu’s harmonic mean Equation (3):

(1) $$\gamma_{SL}+\gamma_{LV}cos\theta =\gamma_{SV}$$

(2) $$\gamma_{SV}=\gamma_{SV}{}^{p}+\gamma_{SV}{}^{d}$$

(3) $$\gamma_{SL}=\gamma_{SV}+\gamma_{LV}-4\left[\frac{\gamma_{SV}{}^{d}\gamma_{LV}{}^{d}}{\gamma_{SV}{}^{d}+\gamma_{LV}{}^{d}}+\frac{\gamma_{SV}{}^{p}\gamma_{LV}{}^{p}}{\gamma_{SV}{}^{p}+\gamma_{LV}{}^{p}}\right]$$

In these equations, γ refers to the values of the surface energy and the letter of S, L and V represent the solid, liquid and vapor, respectively. The subscripts d and p represent the dispersive part and the polar part of the surface energy respectively. θ is the contact angle between the liquid and solid surface. 

The interfacial tension between clays and virgin PA6 was determined through the geometric mean Equation (4):

(4) $$\gamma_{12}=\gamma_{1}+\gamma_{2}-2\left[{\left(\gamma_{1}{}^{d}\gamma_{2}{}^{d}\right)}^{1/2}+{\left(\gamma_{1}{}^{p}\gamma_{2}{}^{p}\right)}^{1/2}\right]$$

In Equation (4), γ_12_ is the interfacial tension between composite 1 and 2, while γ_1_ and γ_2_ are the surface energy of 1, 2 respectively.

Thermogravimetric analysis (TGA) was carried out by a Q-50 instrument (TA, Co., Chicago, IN, USA) at a heating rate of 10 °C/min from ambient temperature to 800 °C under nitrogen flow. The samples were maintained at about five milligrams and were measured with three replicates.

The LOI value was obtained using a JF-3 oxygen index meter (Jiangning Analytical Instrument Company, Jiangning, China) with the dimension of all samples being 80 × 10 × 4 mm. This is conducted according to ISO 4589-1984. 

The UL-94 vertical burning test was performed according to on an SH5300 instrument (Guangzhou Xinhe Electronic Equipment Co., Ltd., Guangzhou, China) with samples according to ASTM D3801-2010. Samples with dimensions of 130 × 10 × 3.2 mm and 130 × 10 × 1.6 mm were tested.

The combustion behavior of the composites (10 × 10 × 6 mm^3^) were measured with a cone calorimeter measurement at an incident heat flux of 50 kW/m^2^ according to ISO5660-1.

The morphology of the burning residue after cone colorimeter tests, including outer surface, inner surface and inner layer of the residue, was observed using the scanning electronic microscopy (SEM, KYKY Technology Development Ltd., Beijing, China) operating at an accelerating voltage of 10 kV. The chemical composition of char residue was characterized by the energy dispersive X-ray spectroscopy (EDS, INCA-350, Oxford, UK).

Dynamic mechanical properties were analyzed using dynamic mechanical analysis (DMA, Q800, TA Co., Chicago, IN, USA) under the dual cantilever tensile mode at a frequency of 1 Hz and a heating rate of 5 °C/min from 20 °C to 160 °C.

## 3. Results and Discussion

### 3.1. Microstructure Characterization of PA6 Composites

#### 3.1.1. XRD Analysis

**Figure 1:**
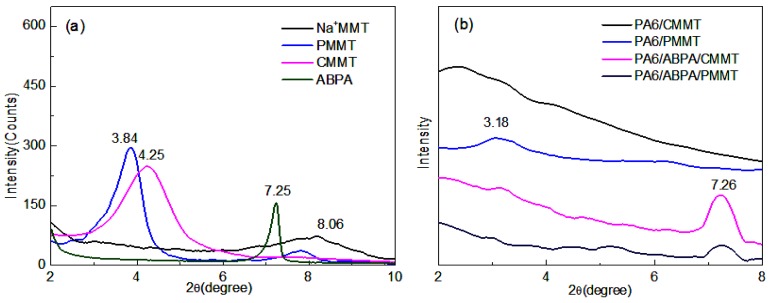
XRD spectra for (**a**) OMMT and flame retardant; (**b**) PA6 nanocomposites.

The XRD spectra of MMTs and their nanocomposites are shown in Figure 1. From the angular location of the peaks and Bragg equation, the *d*_001_ spacing for each MMT and a series of corresponding nanocomposites can be calculated. From Figure 1a, the peak of the 001 plane for pristine MMT is at 2θ = 8.06° (corresponding to the *d*_001_ spacing *d* = 1.1 nm), while the diffraction peaks for CMMT and PMMT samples appear at 2θ = 4.25° (*d* = 2.1 nm), and 3.84° (*d* = 2.3 nm) respectively. The results indicate that quaternary phosphonium salt with a bulkier molecular structure is interacted into the MMT interlayer, and has a greater impact on the interlayer spacing of MMT than quaternary ammonium salt. This effect occurs because the alkyl group in quaternary phosphonium salt is longer and the radius of the center phosphorus atom is larger than that of the nitrogen in CMMT [22]. For the PA6/OMMT nanocomposites shown in Figure 1b, the 001 diffraction peak for PA6/PMMT shifts toward 2θ = 3.18°, corresponding to the *d*_001_ spacing of PMMT increasing slightly to 2.8 nm after compounding with PA6, which implies that a stacked intercalated structure exists. In comparison, for the PA6/CMMT nanocomposite, there is no significant peak in the range of 2θ = 2–5° but peak breadth increases, suggesting a mixed intercalated-exfoliated structure. It is interesting to note that the spectra of both PA6/ABPA/OMMT nanocomposites have no peaks in their diffraction patterns except for the characteristic peak of ABPA at 7.26°. It can be said that a higher degree of exfoliation of the MMT layers was obtained in the PA6/ABPA/OMMT nanocomposites. 

#### 3.1.2. TEM Analysis

**Figure 2:**
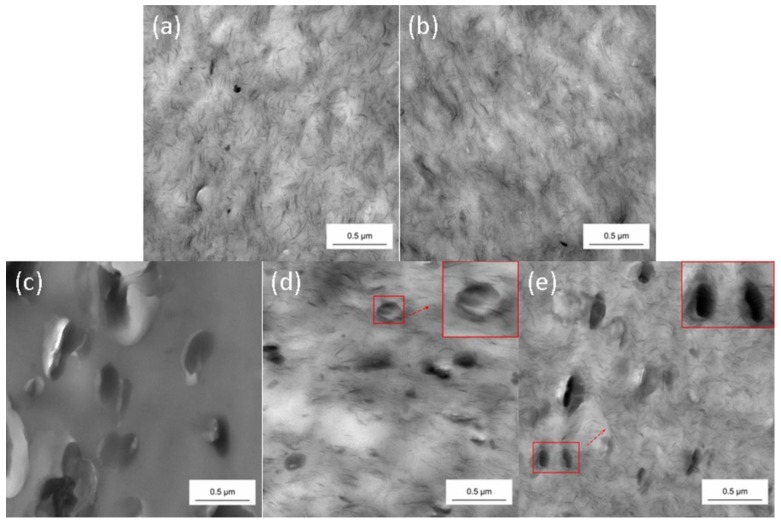
TEM images of (**a**) PA6/CMMT; (**b**) PA6/PMMT; (**c**) PA6/ABPA; (**d**) PA6/ABPA/CMMT; (**e**) PA6/ABPA/PMMT, in which the dark lines represent the clay platelets and the gray areas present the flame retardant particles.

**Scheme 2:**
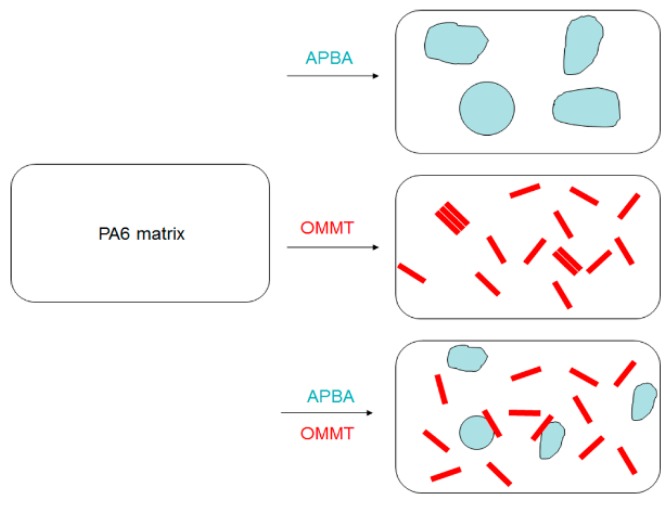
The schematic illustrations for different PA6 composites.

TEM was used to investigate the dispersion of the clay in PA6/OMMT and PA6/ABPA/OMMT composites (Figure 2). In the PA6/CMMT blends, it is clear that most clay platelets are exfoliated in the PA6 matrix, and some intercalated structures are also observed (Figure 2a). This indicates a mixed intercalated-exfoliated structure, which is consistent with the XRD results. In the PA6/PMMT blends, stacked intercalated structure and single exfoliated layers coexist. However, in the PA6/ABPA/OMMT blends, almost all clay platelets are exfoliated and few intercalated structures can be observed independently of the type of OMMT. The improved dispersion of OMMT in flame retardant samples can be interpreted as due to the stronger interaction between PA6 and clay after the incorporation of ABPA. From Figure 2d,e, clay platelets are located in the PA6 matrix as well as at the interface of PA6 and ABPA. Besides, the domain size of ABPA become smaller after the introduction of OMMT. It can be explained by the accumulation of clay at the interface, which prevents the coalescence of the ABPA domain [23]. The improved dispersion of flame-retardant particles in flame-retardant nanocomposites after the introduction of clay platelets has been observed in previous studies. In Cao et al.’ studies, the incorporation of OMMT into TPV/MH composites improved the dispersion of MH particles in the polymer matrix and induced the formation of a clay network [24]. TEM analysis together with XRD results demonstrate that the dispersion of ABPA particles and OMMT platelets are both improved with the incorporation of both. The schematic illustration of the microstructure is presented in Scheme 2.

Regarding the dispersion and distribution of clay platelets in flame-retardant polymer system, various micromorphologies have been reported [25,26]. Fang et al. compared the dispersion and distribution of clay platelets in PP/BER-AO/OMMT and PP/PP-*g*-MAH/BER-AO/OMMT composites. For PP/BER-AO/OMMT, OMMT platelets were aggregated in the BER-AO domains. With incorporation of the compatilizer PP-*g*-MAH, OMMT platelets were dispersed in the PP matrix, which were more efficient at stabilizing the polymer and preventing aggregation of BER-AO during burning, resulting in improved flame retardancy [25]. Similarly, Lu et al. investigated the influence of dispersion of clay in APP/ABS/PA6 blends with disperse PA6 phase [23]. The results demonstrated that when clay dispersed both in the PA6 phase and at the interface between PA6 and ABS, the reinforcement of clay platelets on residue char and the improvement of flame retardancy was more significant compared to the dispersion of clay only in the PA6 or at the interface. These results demonstrate that the dispersion and location of clay platelets in polymer matrix play an important role on the final flame retardancy. Therefore, we hypothesize that the even dispersion of clay platelets in the whole system in the present study may contribute to the formation of thick and uniform char layers at the end of combustion.

### 3.2. TG Analysis

**Figure 3:**
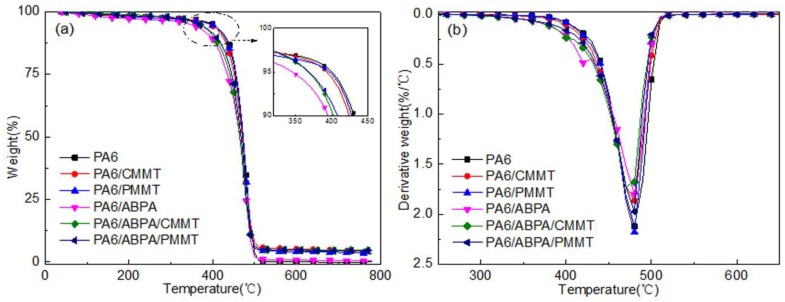
TG (**a**) and DTG (**b**) curves of PA6 and PA6 composites under nitrogen atmosphere on heating ramp of 10 °C/min.

Thermal decomposition of PA6 and PA6 composites in a N_2_ atmosphere was analyzed by TGA, and the results are shown in Figure 3 and listed in Table 2. The pure PA6 begins to decompose at 400.6 °C and the *T*_max_ appears at 479.8 °C with a maximum weight loss rate of 2.1%/min. The residue is 0.2 wt % at 800 °C and the thermal degradation process is only one step associated with the thermal decomposition of PA6 [27]. With the incorporation of organoclays into PA6, the initial decomposition temperatures of PA6/CMMT and PA6/PMMT slightly reduce to 393.7 °C and 397.4 °C, respectively, which is attributed to thermal decomposition of surfactants of the organoclays [28]. The residue of PA6/CMMT and PA6/PMMT composites increase from 0.2% to 3.7% and 3.6%, respectively. Furthermore, the maximum degradation rate is lower than pure PA6 resin, showing an enhanced thermal stability due to the barrier properties of MMT layers. The addition of 12 wt % ABPA to PA6 significantly decreases the *T*_onset_ to 345.7 °C, and the final residue at 600 °C increases to 0.7%. The decreased *T*_onset_ can be attributed to the early decomposition of ABPA to produce alkyl segments, alkyl gases, CO_2_, and alkyl-substituted phosphinates [29,30]. Furthermore, the thermal decomposition of PA6/ABPA consists of two inconspicuous steps according to the two peaks in the DTG curve, which can be assigned to the degradation of ABPA and the backbone of PA6, respectively. Similar two mass-loss stages are observed for PA6 flame retarded by the mixtures of aluminium salts of diisobutylphosphinic acid and monoisobutylphosphinic acid. The first weight loss stage is attributed to the inherent properties of the flame retardant [26]. With the incorporation of OMMT into the PA6/ABPA, *T*_max_ merges into one peak, which can be associated with the improved dispersion of ABPA in the PA6/OMMT/ABPA composite. For PA6/ABPA/CMMT and PA6/ABPA/PMMT composites, the *T*_onset_ increases by 22.0 and 27.8 °C, and the amount of residue at 600 °C increases by 4.0% and 4.9% respectively, compared to that of PA6/ABPA. This enhanced thermal stability is attributed to the formation of a physical protective barrier created by the ablative reassembling of the silicate layers, which delays the diffusion of volatile decomposition products [31].

### 3.3. Viscoelastic Measurements

**Figure 4:**
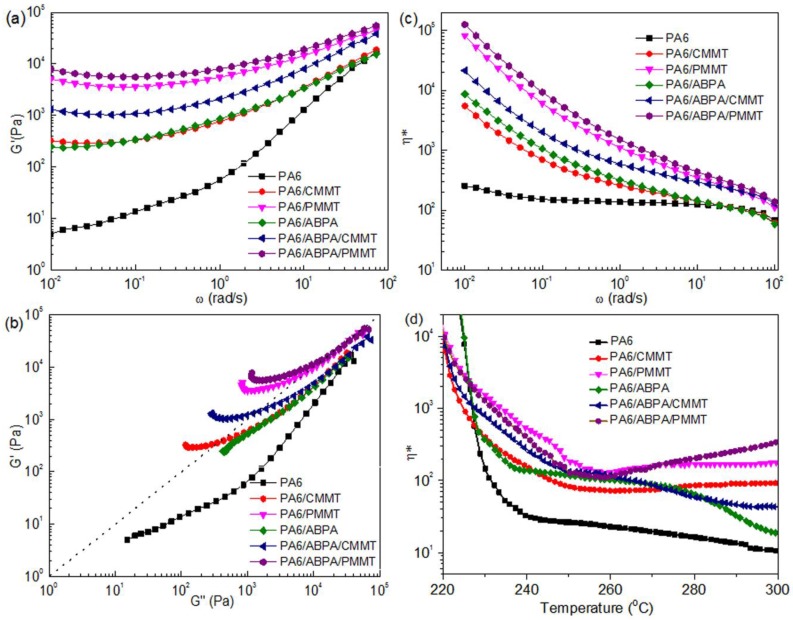
Storage modulus (**a**); melt viscosity (**b**) and Han polt (**c**) versus frequency at 230 °C, and melt viscosity (**d**) versus temperature at 0.1 rad/s for PA6 and flame retardant PA6 composites.

Figure 4a shows the storage modulus (*G*’) of the PA6 and PA6 composites with different organoclays at 230 °C. In the high ω regime (>10 rad/s), all samples show the same trend and there are no significant differences in the slopes of the curves. However, the slope of log *G*’ versus log ω plot in the low frequency range (<0.1 rad/s) is extremely small after the addition of clay, even starts to develop a plateau, especially for PA6/ABPA/PMMT, which is generally the pseudo-solid like behavior of the materials at the terminal region [32]. Furthermore, the incorporation of OMMT increases the *G*’ values at the low frequency range which follows the order of: PA6 < PA6/ABPA < PA6/CMMT < PA6/ABPA/CMMT < PA6/PMMT < PA6/ABPA/PMMT. The results reveal that the flame-retardant nanocomposites show higher *G*’ values than the corresponding PA6/OMMT nanocomposites without flame retardants and the highest *G*’ value is observed for PA6/ABPA/PMMT. 

Similar trends are observed for the complex viscosity (η*) of PA6 and PA6 nanocomposite (Figure 4b). The η* of the PA6 shows almost Newtonian behavior, while all the other samples exhibit a very strong shear-thinning tendency in low ω regime. The PA6/ABPA/PMMT presents the highest η* value in the frequency range. This suggests that a stronger network structure is established in the sample of PA6/ABPA/PMMT since the change of dynamic modulus and viscosity in low ω regime are characteristics for the formation of network-like structures. On one hand, the clay network can improve the melt viscosity and limits the mobility of the polymer chains during combustion. On the other hand, the network structure can efficiently improve the barrier character to the evolution of flammable volatiles and the ingress of oxygen to the condensed phase, leading to a significant improvement in flame retardancy of the nanocomposites [18,33].

The terminal behavior of the PA6 and its composites can also be illustrated in the Han plot (log *G*’—log *G*”, Figure 4c). The diagonal line (*G*’ = *G*”) divides the figure into two parts and determines the transition from viscous behavior (corresponding to *G*’ < *G*”) to elastic behavior (corresponding to *G*’ > *G*”) [34]. The Han plots of pure PA6 and PA6/ABPA composite were located below the diagonal line that behaving a viscous response. The incorporation of organoclays induces a visco-elastic transformation, as evidenced by the curves passing across the diagonal line. It is important to note that the crossing point between the curve and diagonal line shifts to a higher frequency for PA6/ABPA/CMMT and PA6/ABPA/PMMT nanocomposites with exfoliation structures than PA6/CMMT, PA6/PMMT nanocomposites, respectively, due to the increased physical association within the composite systems at the better nanodispersed clay layers [35].

The above viscoelastic measurements were conducted at 230 °C. However, the hottest regions of the samples would be in the temperature range of 350–400 °C during burning. Accordingly, the persistence of the network structure at higher temperatures becomes critical for effective flame retardancy [17]. Therefore, viscoelastic measurements were also conducted from 230–300 °C at a frequency of 0.1 rad/s, since some of the composite samples start to degrade slowly at around 300 °C and the results are shown in Figure 4d. The η* of PA6 sharply decreases at first, and then gradually decreases with an increase in temperature. The *G*’ of PA6/ABPA shows a similar trend and is slightly higher than that of PA6. With the incorporation of OMMT alone, the η* value decreases and then remains nearly constant with an increase in temperature. The highest η* value is observed for PA6/ABPA/PMMT at 300 °C. The trend indicates that the network structure in PA6/ABPA/PMMT would be preserved at high temperature, probably even during burning.

A similar improvement is observed in the storage moduli of the PA6/clay nanocomposites, which is ascribed to the combined effect of aspect ratio and degree of dispersion of the clay particles [36]. However, this raises the question of why an increased melt viscosity is obtained for PA6/ABPA/PMMT. Several possible reasons are considered regarding the higher viscosity for PMMT-containing system compared to the CMMT-containing system: the factual content of MMT in polymer matrix, the dispersion state of MMT platelets, and the interfacial interaction. From XRD and TEM, clay platelets with similar exfoliation are observed in two OMMT systems and the dispersion state of clay platelets is almost the same. Residues at 800 °C from TG reveal that the clay contents in PA6/ABPA/CMMT and PA6/ABPA/PMMT are 4.7% and 4.5%, respectively, with no obvious difference observed. Regarding the interfacial interaction, the surface tensions of the neat PA6 and clays were calculated based on Equations (1)–(3) in the experimental sections at room temperature, before the results were extrapolated to processing temperature according to temperature coefficients (−dγ/d*T*) obtained from literature [37,38,39]. The interfacial tensions between the neat PA6 and clays were further calculated using the geometric mean Equation (4). The results are listed in Table 3. The interfacial interaction between PA6 and CMMT is even lower than that of PA6 and PMMT from Table 3, suggesting a better compatibility for PA6 and CMMT.

Therefore, it is reasonable to speculate that the lower viscosity and modulus for the CMMT-containing system may be due to the more significant plasticizing effect of the clay modifier in CMMT [40]. It is reported that although the organic intercalation can increase the compatibility between clay and polymer matrix, the interaction of the modified clay layers with polymer matrices remain as weak as van der Waals interactions. Besides, adverse plasticization effects at the clay—matrix interface may exist [40,41]. 

**Figure 5:**
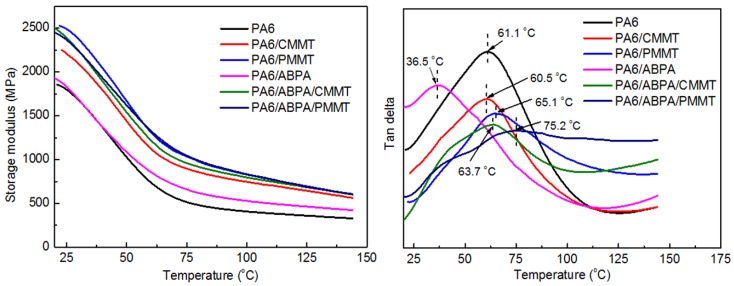
Dynamic storage modulus (**a**) and tan δ (**b**) vs. temperature for PA6 and various PA6 composites.

To confirm our speculation, the Dynamic Mechanical Analysis (DMA) was conducted, with a special focus on glass transition temperature (*T*g) since it has been repeatedly reported the plasticization effect would reduce *T*g. Figure 5a illustrates the DMA plots of storage modulus versus temperature for various systems and *T*g can be obtained from temperature corresponding to the maximum value of tan δ (Figure 5b). As seen from Figure 5, the incorporation of ABPA increases the storage modulus at *T* > *T*g, which is the rubbery region of PA6 system. In contrast, the addition of 6 wt % clay increases storage modulus in both glassy and rubbery regions at all temperatures independently of the presence of flame retardant ABPA. The storage modulus of PMMT-containing system is higher than CMMT-containing system. This is due to the formation of a more significant plasticization effect on PA6 at the interface with CMMT. This leads to a reduction in the ability of load transfer from matrix to the CMMT, resulting in a lower *T*g of CMMT-containing system in comparison to PMMT-containing system (Figure 5b). As presented, *T*g of the PA6 presents no obvious change when incorporating CMMT into PA6, which is consistent with previous reports [42]. The *T*g of PA6 /PMMT shows about 4 °C and 5 °C increases, compared to the pure PA6 and PA6/CMMT, while the *T*g of PA6/ABPA/PMMT shows about 14 °C and 9 °C increases, respectively, compared to the pure PA6 and PA6/ABPA/CMMT. These increments demonstrate that there is a smaller plasticization effect present at the interface of PMMT and PA6, and show that PMMT can establish a strong interface with PA6, leading to the restriction of segmental chains motion. In conclusion, the higher *G*’ and viscosity for PMM-containing samples compared to CMMT-containing samples is attributed to the decreased plasticization effect. Furthermore, other factors cannot be excluded, such as the molecular weight of the matrix or the aspect ratio of the dispersed clays [43].

### 3.4. Flame Retardancy Performance

#### 3.4.1. UL-94, LOI and Cone Calorimetric Results

The influence of different organoclays and ABPA on the flame-retardant properties of PA6 composites was evaluated by LOI and UL-94 vertical burning tests. The relevant test results are listed in Table 4 and Table S1. Since the combustion is long-lasting and accompanied by severe dripping, PA6 could not be rated in term of UL-94. The LOI value decreases to 21% and 24.2%, respectively, after incorporating CMMT and PMMT in PA6. This can be explained by the pronounced increase in melt viscosity (as shown in Figure 4b) as revealed by Schartel et al. [44], since heat taken away from the pyrolysis zone by melt is decreased. In the case of PA6/ABPA, LOI value is increased to 30.4% and UL 94 rating reaches a V-0 (3.2 mm) when the loading of ABPA is higher than 12 wt % [19]. When half of the ABPA is substituted with CMMT, the LOI value of PA6/ABPA/CMMT slightly decreases to 29.8% and the UL-94 rating remains at the V-0 ranking (3.2 mm). For PA6/ABPA/PMMT, the LOI value increases to 33.0% from 30.4% for PA6/ABPA. UL 94 V-0 (3.2 mm) is achieved and the total after-flame time (*t*_1_ + *t*_2_) is reduced, suggesting a synergistic effect of ABPA and OMMT, especially PMMT, in improving the flammability of PA6. The samples with thickness 1.6mm were also tested by UL-94 as listed in Table 4 and the results further confirm the good synergism between ABPA and PMMT on the flame retardancy of PA6.

**Figure 6:**
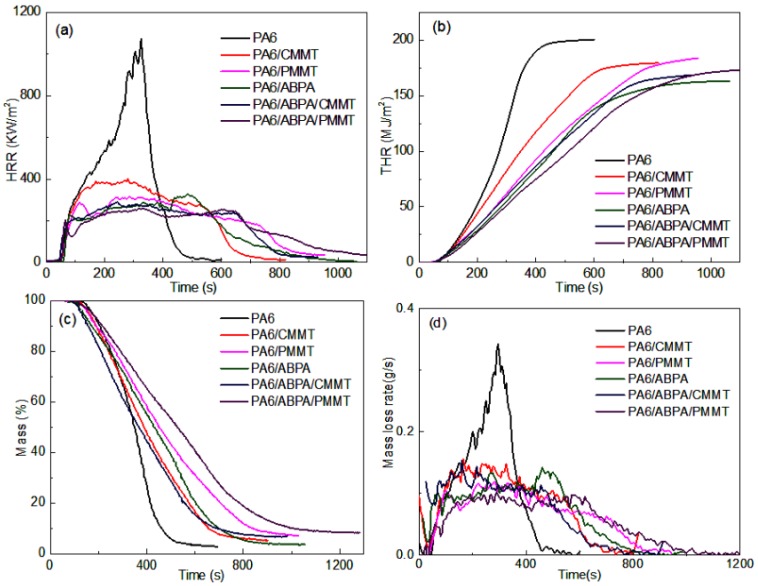
HRR curve (**a**); THR curve (**b**); mass loss curve (**c**) and mass loss rate (MLR) curve (**d**) for PA6 and flame retardant PA6 composites.

To further understand the flame-retardant effect of the ABPA and clays in PA6, the combustion behavior of the PA6 composites was assessed using cone calorimetry. Figure 6a–d illustrate the heat release rate (HRR), total heat release (THR), mass loss and mass loss rate (MLR) curves of all materials, respectively. Detailed data obtained from the cone calorimetry are listed in Table 5. The heat release rate, especially peak HRR, has been considered to be an essential parameter in evaluating fire hazard [45]. Compared to virgin PA6, the HRR of PA6 composites is decreased to different extent and burning time is extended, and the plateau stages in HRR curves which are caused by char forming are observed. The PHRR values of PA6/CMMT and PA6/PMMT are 62% and 71% lower than that of pure PA6, respectively. With the incorporation of 12% ABPA, the PHRR value decreases by 69%. The PHRR is further reduced by about 73% in PA6/ABPA/CMMT and 75% in PA6/ABPA/PMMT in comparison with the PA6 when ABPA and OMMT are both present. The AHRR values show a similar trend to the PHRR values, with the lowest PHRR and AHRR values obtained for PA6/ABPA/PMMT composites.

The incorporation of CMMT or PMMT slightly decreases the THR values due to the substitution of the organic polymer matrix with inert fillers (dilution of fuels in the condensed phase). The THR value decreases from 200.3 for PA6 to 163 with incorporation of 12 wt % ABPA, due to the significant fire inhibition effect of flame retardant [19]. The THR is slightly increased when ABPA is partially substituted with OMMT.

A slight decrease in effective heat of combustion (EHC), which is determined by dividing the heat release rate by the mass loss rate, is observed for PA6/OMMT nanocomposites, while greater decrease is found from 27.7 for PA6 to 22.5 for PA6/ABPA. Similar to THR, the EHC is slightly increased when ABPA is partially substituted with OMMT.

**Figure 7:**
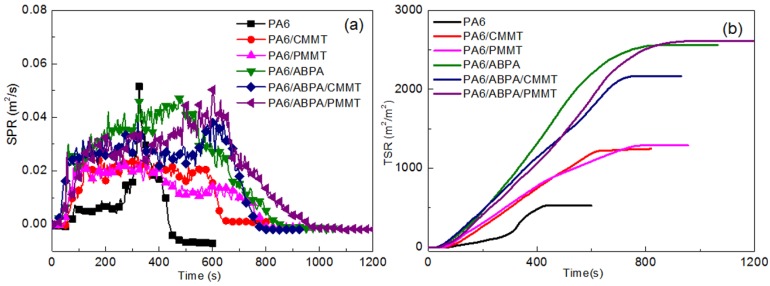
Smoke production rate (SPR, (**a**)) and total smoke production (TSP, (**b**)) plots of PA6 and PA6 composites.

**Figure 8:**
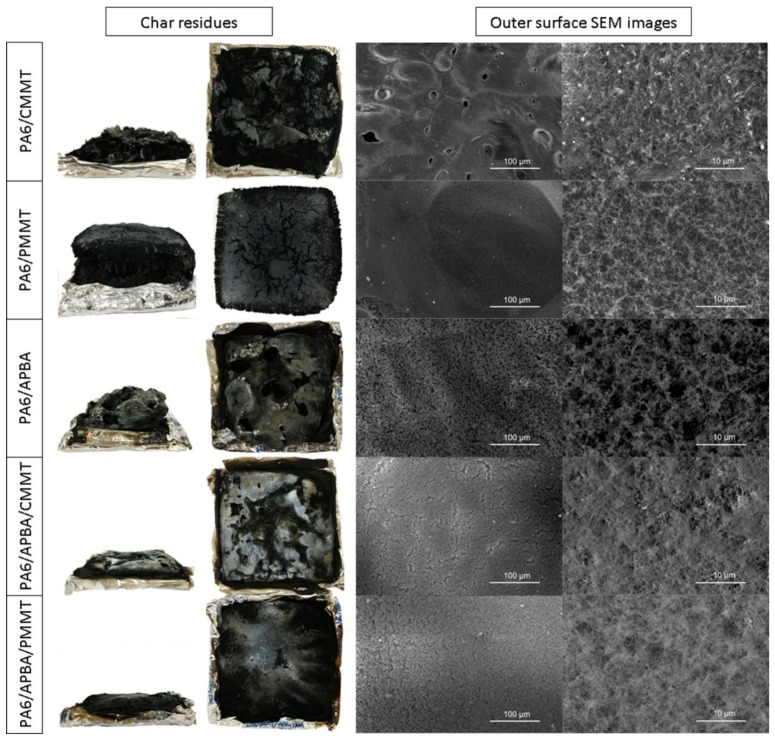
Photographs (the left two rows) and SEM images at different magnifications (the right two rows) of the residues after the cone calorimeter tests for PA6 and PA6 composites.

In Figure 7 smoke production parameters are reported. The incorporation of OMMT increases the Smoke Production Rate (SPR) and the Total Smoke Production (TSP) due to increased char layer, resulting in more incomplete combustion. The PA6/APBA sample show more significant increase in SPR and TSP, with TSP increasing from 528.58 m^2^/m^2^ for PA6 to 2547.06 m^2^/m^2^. The increased TSP together with decreased EHC reflects the gas phase activity of ABPA. For PA6/ABPA/CMMT, the SPR and TSP slightly decrease due to the decreased APBA loading, while for PA6/APBA/PMMT, the SPR and TSP are similar to that for PA6/APBA. It is probably caused by two competitive factors: on one hand, the decreased APBA loading leads to decreased smoke release; on the other hand, the compact char layer (as shown in Figure 8) prevents the complete oxidation of degradation products, resulting in the release of only partially oxidized carbonaceous residues and more smoke release.

Regarding the residue weight after burning, it is noticed that the residue weight of PA6/CMMT and PA6/PMMT composite was 5.2 and 7.1, respectively, compared to 2.7 for neat PA6, suggesting a better charring effect in the case of PMMT. Comparably, incorporation of 12 wt % ABPA increases the char residue to 3.7 and the char residues further increase to 6.9 and 8.2 when ABPA is partially substituted with OMMT. These values are slightly higher than that of PA6/CMMT and PA6/PMMT. Based on the mass loss curves obtained in the cone calorimeter (Figure 6c,d), the mass loss rate of neat PA6 is slowed with the addition of ABPA or OMMT because the bits of residue or the accumulation of clay platelets at the upper surface hinder the release of volatiles [28]. The combination of OMMT with ABPA in the flame-retardant PA6/ABPA composites further decreases the mass loss rate due to the thick, compact, and continuous char layer formed by migration and accumulation of clay layers (Figure 8), especially in the case of PA6/ABPA/PMMT.

#### 3.4.2. Char Residue Analysis

To understand the flame-retardant mechanism of different PA6 composites, the structure of the residue char after burning was investigated. From the photographs presented in Figure 8, broken char residues are observed for PA6/CMMT, while continuous and intumescent char layer with some cracks on the surface are observed for PA6/PMMT. After comparing the amount of the char residue for the two samples, we find that the amount of the residue for PA6/PMMT (7.1%) is slightly higher than that of PA6/CMMT (5.2%). The results suggest that the strength of the char layer for PA6/CMMT is not big enough to afford the weight, resulting in collapse of the char layer. However, for PA6/PMMT, the high strength can resist the vigorous bubbling of evolved degradation products during combustion, which allows for the formation of a continuous char layer with some cracks. Besides, the intumescent char layer for PA6/PMMT is foam-like, which is similar to the structure of protective layer generated by the combustion of intumescent flame retardant (IFR) system [46]. Generally, the char forming agent in the IFR will swell during combustion to form intumescent charring layer with many mini-pores, which in turn acts as a barrier to heat and mass transfer and protects inner matrix materials. Similarly, the foam-like structure of char layer for PA6/PMMT can also provide a better barrier for heat transfer, which is consistent with the PHRR results. For PA6/ABPA, a slightly intumescent char layer with big holes on the surface is formed. With the substitution of half of ABPA with CMMT, the char layer becomes compact with decreases in the size and number of holes on the surface. Comparably, a more compact and continuous char layer is observed for PA6/ABPA/PMMT. 

In order to elucidate the relationship between the microstructure of the charred layer and flame retardance of the sample, we characterized the char morphology after burning by SEM (Figure 7). For the PA6/CMMT sample, it can be observed that there were many small holes on the char surface after burning, which can provide routes for heat and mass transfer between the polymer under the burning surface and the air. Therefore, this is not beneficial for improving flame retardancy. In contrast, fewer big cracks or holes can be observed on the surface of the PA6/PMMT samples after combustion. The image at higher magnification shows that the char layer is composed of network structures. This can be correlated with the increased melt viscosity. For the PA6/ABPA sample, a continuous char layer with many interconnected tiny holes was observed after combustion due to adequate loading of flame retardant ABPA, which is similar to that reported by Wang et al. for the PA6/GF/ABPA system [47]. The substitution of half of ABPA with OMMT in the composites results in a different char morphology. For PA6/ABPA/CMMT composites, many strip cracks are observed on the char surface after combustion. In contrast, no obvious cracks are observed and more compact structure compared to PA6/ABPA is formed for PA6/ABPA/PMMT composites.

**Figure 9:**
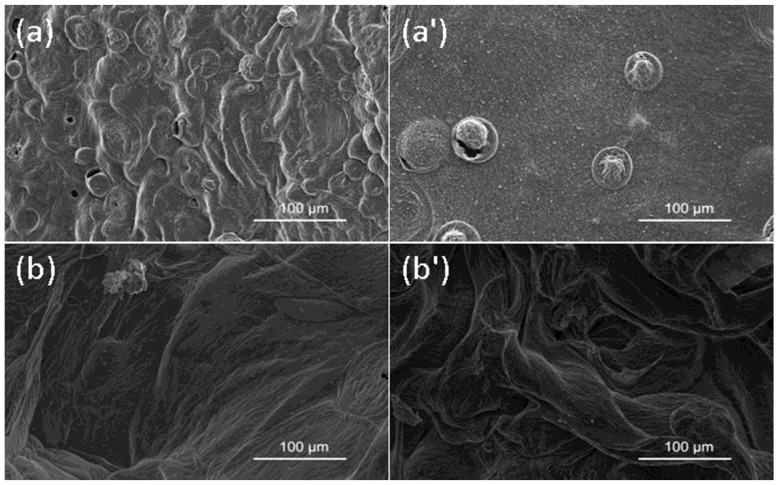
The Inner surface (left row) and Inner layer (right row) for PA6/ABPA/CMMT (**a**,**a’**) and PA6/ABPA/PMMT (**b**,**b’**).

To conduct a detailed analysis of the residue microstructure for the flame-retardant composites, the residue char layer was carefully trimmed to expose the inner surface and inner layer of the char layer. The inner surface and inner layer was characterized by SEM with the results displayed in Figure 9. For PA6/ABPA/CMMT composites, many spherical bubbles are observed in the inner surface and some are broken. A similar structure is observed in the inner layer and the number of bubbles decrease. 

Different from the residue microstructure of PA6/ABPA/CMMT composites, drastic expansion is observed, and no small bubble is detected for PA6/ABPA/PMMT. Moreover, the inner surface is curved with no cracks, which means that the surface was covered by the full char residue. The crimp and overlapping are more significant in the inner layers. When we combine these observations with the results in Figure 4, it is rational to attribute the different burning behaviors of the two samples to the significantly higher melt viscosity of the samples containing PMMT. The degradation of the polymer matrix during burning generates many gas bubbles inside the sample. These bubbles rise and expand to the sample surface when the surrounding polymer melt has a low viscosity, which is the case for the PA6/ABPA/CMMT sample. Therefore, we observed many holes at the char surface of the sample. Obviously, these holes will transfer the mass and the heat during burning. In contrast, with the addition of PMMT, the melt viscosity of the sample increases, as shown in Figure 4. The high melt viscosity suppresses the vigorous bubbling and the rising of the formed bubbles. Therefore, no holes are observed, and a moderate swelling of the sample is observed. Consequently, the burning surface stays compact, continuous, and dense with no cracks after burning.

#### 3.4.3. EDS Analysis

The superior fire performance of polymer/clay nanocomposites is mainly a consequence of an ablative reassembling of the clay platelets on the surface of the burning nanocomposites, which creates a physical protective barrier [23]. The critical factors in determining their FR effectiveness include the accumulation of clay platelets near the sample surface and their area coverage over the degrading sample surface [28]. Table 6 shows the elemental contents at the outer surface of the char layer. The accumulation of clay on the outer surface of char residue is observed for both PA6/CMMT and PA6/PMMT. Comparably, the C mass ratio of PA6/CMMT residue is lower and the Si and O mass ratios are higher than that of PA6/PMMT residue. It reveals that a great C content is locked in the condensed phase in PA6/PMMT composites. This is due to the more efficient barrier effect of clay platelets that decelerates heat and mass transfer between the gaseous and condensed phase. Additionally, the P mass ratio is almost negligible for PA6/PMMT composites, suggesting that the P in the intercalating agent does not attribute to the condensed phase flame retardancy mechanism. For PA6/ABPA, high P and Al mass ratios and a moderate C mass ratio are observed on the residue surface due to the incorporation of ABPA. In flame-retardant nanocomposites, additional P and Al elements at the outer surface of char residue are observed. In comparison, the C and Si mass ratios for PA6/ABPA/PMMT are higher than that for PA6/ABPA/CMMT, while the Al and P mass ratios are lower.

#### 3.4.4. Flame-Retardant Mechanism Analysis 

According to our previous work, the flame retardant ABPA works mainly in the gas phase by flame inhibition, which is accompanied by a minor effect on the condensed phase by forming bits of residues. With the introduction of OMMT, the amount and thermal stability of the char layer increases, and condensed-phase flame-retardant effect is enhanced [19]. In this study, the type of the OMMT mainly affects the melt viscosity and the microstructure of the char layer after combustion. For PA6/OMMT nanocomposites, network structures are formed in the initial samples and the measured storage and viscosity significantly increases compared to pure PA6. As the temperature of the sample becomes high enough during burning, degradation of PA6 occurs and a thin layer is formed due to the migration of silicates on the surface of the burning materials [48,49]. The degradation products form bubbles in the melt polymer and rapidly rise to the sample surface. The melt viscosity of the sample containing CMMT is not large enough to fix the bubbles and the bubbles burst at the surface, resulting in the formation of cracks or holes going through the residue [17]. These cracks are pathways of gas fragments generated from the combustion and heat evolved during burning process. Correspondingly, poor flame retardancy is exhibited in PA6/CMMT. For PA6/PMMT nanocomposites, an adjusted viscosity needed for efficient intumescence is obtained. The viscosity is small enough for the residue to expand, and large enough to fix the bubble in a foam-like structure, resulting in the formation of efficient intumescence [23]. In blends of PA6/ABPA/OMMT in which the clay is located in PA6 and at the interface, the ABPA domain decreases. More efficient network structures are formed and the measured storage and viscosity is higher than that of the corresponding PA6/OMMT composites. For PA6/ABPA/CMMT, the viscosity is not big enough, and the volatiles in terms of bubbles could agitate the melted polymer substrate and interfere with the formation of a compact and continuous char layer. In contrast, the viscosity of PA6/ABPA/PMMA in elevated temperature is high enough to behave like a solid material during combustion, which can retard the motion of polymer chains more efficiently during thermal degradation. At the same time, the bubbles remain small in the higher viscosity layer and their transport to the surface tends not to disrupt the structured layer which allows for the preservation of the char layer during combustion. Due to the increased viscosity inhibiting the formation of large bubbles and then the swell of the PA6 matrix, the swell of the intumescent char is decreased compared to PA6 /PMMA (as shown in Figure 8). Compared with PA6/ABPA/CMMT, the measured viscosity of PA6/ABPA/PMMT in elevated temperature is higher mainly due to the decreased plasticization effect at the interface between PMMT and PA6. Correspondingly, a continuous protective shield is formed on the slightly intumescent char. As a result, PA6/ABPA/PMMT exhibits the best flame retardancy in all tested samples. Regarding the effect of viscosity on the char expansion, previous studies have observed a similar phenomenon [50]. Clerc et al. showed that materials (EVA containing lamellar particles) with an average viscosity (neither too low, nor too high), present the highest cone calorimeter residue thickness after burning. Such a viscosity enables to trap bubbles and thus create a swollen structure. This has also been shown in other studies concerning the ABS/PA6 fire behavior [23]. If the viscosity is too high, bubbles will not spread and grow inside the material. Consequently, the final thickness of the cone calorimeter decreases. 

**Figure 10:**
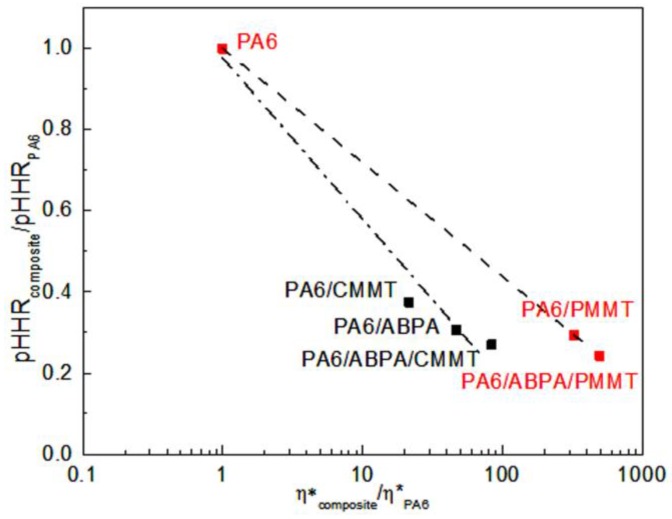
Variation of reduced pHRR as a function of increased viscosity at low frequency for PA6 and PA6 composites.

To quantitatively investigate the effect of viscosity on the fire performance, the correlation between sample viscosity and cone calorimeter results (Table 5) was examined using the representation proposed by Batistella et al. [51]. The reduced PHRR was plotted against the increased viscosity at a low angular frequency of 0.01 rad/s. As illustrated in Figure 10, for both the CMMT-containing systems or the PMMT-containing systems, the pHRR decreases linearly with the logarithm of the increased viscosity. The best fire behavior is obtained for PA6/ABPA/PMMT, which is the most viscous composite at the low angular frequency (η* = 1.3 × 10^5^ Pa·s), which is probably due to the stronger inter-particles interactions at submicron scale (formation of a stronger network). A similar trend was observed in previous studies when using silicas in polypropylene [52] or polybutylene terephthalate [53].

Previous studies have demonstrated that the nanomorphology of clay (exfoliation, intercalation and presence of tactoids) does not play any significant role to obtain in improving flame-retardant properties if the nanodispersion of clay is achieved [54,55]. Considering that the nanomorphology of clay is similar in PA6/ABPA/PMMT and PA6/ABPA/CMMT, it can be concluded that the main difference between CMMT and PMMT lies in the plasticization effect at clay-matrix interface. PA6/APBA/PMMT presents better flame retardancy than PA6/APBA/CMMT due to the decreased plasticization at the interface between PMMT and PA6 and the formation of more efficient network structure, and then the formation of more compact char layer, resulting in better flame retardancy.

## 4. Conclusions

Two different organoclays were combined with ABPA to improve the flame retardancy of PA6 and the effect of organoclays on the structure of untested samples and char morphology of the samples after combustion was systematically investigated. The incorporation of both types of OMMTs improves the dispersion of ABPA particles and meanwhile exfoliated structures of clay platelets are obtained for both PA6/ABPA/CMMT and PA6/ABPA/PMMT. Compared to neat PA6, the PA6/OMMT composites present higher storage modulus and viscosity, and storage modulus and viscosity further increase with the combination of ABPA and OMMT. Furthermore, the storage modulus and viscosity are higher for PA6/ABPA/PMMT than for PA6/ABPA/CMMT, due to the decrease plasticization effect at the interface between PA6 and PMMT. The flammability of the flame retardant nanocomposites can be associated with the corresponding rheological behavior and the char layer structure. The PA6/PMMT nanocomposite has moderate viscosity. Thus, the corresponding char layer after combustion presents efficient intumescence, resulting in a more effective protective barrier. On the other hand, PA6/CMMT with a lower viscosity would promote the formation of cracks or holes at the residue surface and thus, the nanocomposite shows a worse flame retardancy. PA6/OMMT nanocomposites additionally modified with flame retardant present higher storage modulus and viscosity compared to the corresponding PA6/OMMT nanocomposites, which results in the formation of compact and effective barrier at the surface after combustion. Comparably, due to the relatively higher viscosity for PA6/ABPA/PMMT even at high temperature, more effective network structures are formed in the untested sample. Consequently, less vigorous bubbling allows for a faster accumulation of clay platelets near the surface of the material to create the protective layer, thus improving the flame retardancy performance. It is proposed that the higher the viscosity at low angular frequency is, the better the fire properties of flame-retardant PA6 nanocomposites are. If polymer–clay nanocomposites could be further made to form more stable network (one possible approach might be decreasing the plasticization effect at the interface between polymer and clay by employing intercalating agents bearing reactive functional groups), the flammability properties of the clay-based polymer nanocomposites could be further improved.

## Figures and Tables

**Table 1:** Compositions of the studied materials in wt %.

Sample	PA6	PA6/CMMT	PA6/PMMT	PA6/ABPA	PA6/ABPA/CMMT	PA6/ABPA/PMMT
CMMT	-	6	-	-	6	-
PMMT	-	-	6	-	-	6
ABPA	-	-	-	12	6	6

**Table 2:** Thermal properties of PA6 and PA6 composites under nitrogen atmosphere.

Sample	*T*_5%_ (°C)	*T*_max_ (°C)	Residue (%)
PA6	400.6 ± 0.1	479.8 ± 0.5	0.2 ± 0.1
PA6/CMMT	393.7 ± 0.2	477.7 ± 0.1	3.7 ± 0.5
PA6/PMMT	397.4 ± 0.7	478.7 ± 0.4	3.6 ± 1.2
PA6/ABPA	345.7 ± 2.1	423.7 ± 0.7/478.4 ± 0.2	0.7 ± 0.1
PA6/ABPA/CMMT	367.7 ± 0.5	475.5 ± 0.3	4.7 ± 0.2
PA6/ABPA/PMMT	368.6 ± 0.1	479.1 ± 0.6	4.5 ± 0.1

**Table 3:** Contact angle measurement results for PA6 and clays.

Sample	Contact Angle (°)		Total Surface Tension at 15 °C (mN/m)	−dγ/d*T* (mN/(m·°C))	Surface Tension at 230 °C (mN/m)			Interfacial Tension (mN/m)
	H_2_O	CH_2_I_2_	γ		γ	γd	γp	γij
PA6	73.5 ± 0.6	42.4 ± 0.2	50.5	0.065 ^a^	36.6	28.3	8.3	-
Na^+^MMT	45.6 ± 0.7	28.1 ± 0.6	68.9	0.43 ^b^	23.5	15.4	8.1	1.94
CMMT	78.3 ± 0.8	35.6 ± 0.2	50.9	0.1 ^c^	29.4	24.3	5.1	0.54
PMMT	86.0 ± 0.2	35.8 ± 0.1	47.7	0.1 ^c^	26.2	23.1	3.1	1.49

^a^ Value for PA6 assumed to be the same as PA 66 reported in ref [37]; ^b^ Value for Na^+^MMT assumed to be the same as kaolin reported in ref [38]; ^c^ Value for organoclays assessed from temperature coefficient of small modifier molecules reported in ref [39].

**Table 4:** LOI and UL 94 tests results of PA6 and PA6 composites.

Sample Composition	LOI (%)	UL 94 (3.2 mm) ^a^			UL 94 (1.6 mm)		
		*t*_1_/*t*_2_ (s)	Dripping	Rating	*t*_1_/*t*_2_ (s)	Dripping	Rating
PA6	25	--	Yes	NR	--	Yes	NR
PA6/CMMT	21	--	Yes	NR	--	Yes	NR
PA6/PMMT	24.2	--	Yes	NR	--	Yes	NR
PA6/APBA	30.4	1.9/3.7	No	V-0	4.2/0.9	Yes	V-0
PA6/APBA/CMMT	29.8	2.3/7.5	No	V-0	10.3/3.8	Yes	V-1
PA6/APBA/PMMT	33.0	0.8/3.6	No	V-0	3.6/4.5	Yes	V-0

^a^ Results are reported as the average value of 5 test bars.

**Table 5:** Cone calorimetric results of PA6 and PA6 composites.

Sample	PHRR (kW/m^2^)	AHRR (kW/m^2^)	THR (MJ/m^2^)	av-EHC (MJ/Kg)	TSP (m^2^/m^2^)	Residue (wt %)
PA6	1074.1	357.5	200.3	27.7	528.57	2.7
PA6/CMMT	402.3	227.2	179.5	24.7	1245.59	5.2
PA6/PMMT	315.1	200.7	183.7	26.3	1287.91	7.1
PA6/ABPA	328.7	160.6	163.0	22.5	2547.06	3.7
PA6/ABPA/CMMT	289.4	189.3	168.5	23.6	2155.98	6.9
PA6/ABPA/PMMT	261.0	150.5	174.6	24.7	2602.36	8.2

**Table 6:** Element composition at the outer surface of char layers for different samples.

	C (wt %)	O (wt %)	Si (wt %)	Al (wt %)	P (wt %)
PA6/CMMT	17.4	59.2	12.2	5.5	-
PA6/PMMT	42.0	42.9	8.4	4.1	0.6
PA6/ABPA	20.3	49.6	-	10.5	18.0
PA6/ABPA/CMMT	15.6	55.6	5.3	11.2	12.6
PA6/ABPA/PMMT	29.7	44.9	7.9	7.8	7.3

## Supplementary Materials

The following are available online at http://www.mdpi.com/2073-4360/10/3/312/s1, Table S1: UL-94 and LOI data of flame retardant PA6 composites.
