# Supplementary material for: Structure and Properties Study of PA6 Nanocomposites Flame Retarded by Aluminium Salt of Diisobutylphosphinic Acid and Different Organic Montmorillonites
Source: Polymers (Basel). 2018 Mar 13;10(3):312. doi: 10.3390/polym10030312 (PMC6415078; doi:10.3390/polym10030312)
Supplement: Supplementary file 1 [file polymers-10-00312-s001.pdf]

Structure and Properties Research of PA6 Nanocomposites Flame Retarded by Aluminium Salt of Diisobutylphosphinic Acid and Different Organic Montmorillonites

Wen-Tao He [1,2], Sheng-Tao Liao [3], Yu-Shu Xiang [1], Li-Juan Long [1], Shu-Hao Qin [1,*] and Jie Yu [1,3,*]

1 National Engineering Research Center for Compounding and Modification of Polymeric Materials, Guiyang 550014, China; wentao.he@usq.edu.au (W.-T.H.); xiang_ys@126.com (Y.-S.X); longlijuanhappy@126.com (L.-J.L.)
2 Center for Future Materials, University of Southern Queensland, Toowoomba 4350, Australia
3 College of Material and Metallurgy, Guizhou University, Guiyang 550025, China; liaosthl@163.com
* Correspondence: qinshuhao@126.com (S.-H.Q.); yujiegz@126.com (J.Y.); Tel.: +135-9504-5554 (S.-H.Q.)

UL-94 and LOI data of flame retardant PA6 composites

Table S1 UL-94 and LOI data of flame retardant PA6 composites.

Sample	composition (wt%)		LOI	UL 94 (3.2mm) [a]		
	PA6	ABPA/AlPi	(%)	t_1/t_2(s)	Dripping	Rating
PA6/ABPA	90	10	28.0	3.2/25.5	Yes	V-1
PA6/ABPA	88	12	30.4	1.9/3.7	No	V-0
PA6/ABPA	85	15	32.2	1.2/5.2	No	V-0
PA6/AlPi	90	10	25.8	>30/--	Yes	Fail
PA6/AlPi	88	12	26.8	>30/--	Yes	Fail
PA6/AlPi	85	15	29.2	0.8/28.9	No	V-1

[a] Results are reported as the average value of 5 test bars.
